# Artificial Intelligence for Automated Analysis and Interpretation of Intravascular Ultrasound in Coronary Artery Disease: A Systematic Review

**DOI:** 10.7759/cureus.117386

**Published:** 2026-10-03

**Authors:** Ashwin Umasankar, Heramba Vigneshwar Sachithananthan, Anusha Chitravanshi, Indrajit Banerjee

**Affiliations:** 1 Medicine, Government Medical College, Thiruvallur, IND; 2 Urology, Oxford University Hospitals NHS Foundation Trust, Oxford, GBR; 3 Medicine, Sir Seewoosagur Ramgoolam Medical College, Belle Rive, MUS; 4 Pharmacology, Sir Seewoosagur Ramgoolam Medical College, Belle Rive, MUS

**Keywords:** ai and machine learning, artificial intelligence(ai), cad: coronary artery disease, deep learning (dl), evidence base medicine, image segmentation, intravascular ultrasound (ivus), ivus, plaque characterization

## Abstract

Coronary artery disease (CAD) leads to considerable morbidity and mortality worldwide. Intravascular ultrasound (IVUS) is an imaging modality to guide percutaneous coronary intervention; however, it requires time-consuming, expert-dependent manual analysis. Artificial intelligence (AI) has been increasingly used for IVUS analysis, but there is limited evidence on its diagnostic accuracy and clinical utility. This review aimed to evaluate the diagnostic performance and clinical usefulness of AI for automated IVUS analysis of coronary arteries. The research protocol was prospectively registered (PROSPERO, CRD420261454806) and followed the PRISMA 2020 guidelines for systematic reviews. PubMed/MEDLINE and Cochrane CENTRAL were searched from inception through July 23, 2026, and July 26, 2026, respectively, for publications assessing AI, machine learning, or deep learning algorithms for the automated analysis of coronary IVUS, including segmentation of structures, identification of plaque types, and quantitative measurements. Two reviewers independently screened and selected eligible studies and extracted the data. The risk of bias was assessed using QUADAS-2 (Quality Assessment of Diagnostic Accuracy Studies-2 (QUADAS-2) and visualized using robvis, and the GRADE approach was used to rate the certainty of evidence. Of the 802 records identified (PubMed, n = 216; Cochrane CENTRAL, n = 586), eight studies met the inclusion criteria and were included in this review. They reported on the performance of convolutional neural networks, U-Net/U-Net++, Efficient-UNet, DeepLabv3+-based models, and gradient boosting in segmenting lumen, vessel, plaque, and stent in coronary IVUS. The Dice similarity coefficient ranged from 0.67 to 0.97, and the intersection over union for segmentation (including Jaccard similarity) ranged from 0.66 to 0.97, with the best performance for lumen and vessel segmentation and the poorest for stent and calcium. The sensitivity and specificity ranged from 62% to 90% and 88% to 99%, respectively, with the correlation to expert assessment generally above 0.9. Overall, the risk of bias was mainly related to patient selection (five out of eight studies) and the reference standard (three out of eight studies), and none of the studies was rated as having a high risk of bias. Due to between-study heterogeneity in the tasks, reference standards, and outcome measures, a meta-analysis was not performed, and the certainty of evidence was rated as low. The current evidence suggests that AI-based methods have good potential for analyzing coronary IVUS with acceptable accuracy, which could be helpful for high-throughput IVUS interpretation in clinical practice. They appear to perform best at segmenting lumen and vessel structures and worst at segmenting stent and calcium. However, due to the overall low certainty of evidence from these mostly retrospective studies, their use should be limited to complementing the work of experts in cardiovascular medicine.

## Introduction and background

Coronary artery disease (CAD) remains one of the leading causes of morbidity and mortality worldwide. Even after major advances in pharmacotherapy, prevention, and coronary revascularization, CAD continues to pose a significant clinical challenge. It is characterized by gradual accumulation of atherosclerotic plaques within the coronary arterial wall, which could result in acute coronary syndromes, heart failure, myocardial ischemia, and sudden cardiac death. Accordingly, the plaque morphology and lesion severity assessment has become important not only for clinical diagnosis but also for individualized treatment planning, risk stratification, and the short- and long-term improvement of cardiovascular outcomes. As the global burden of CAD continues to rise, imaging modalities need to increase in number to provide detailed information about plaque composition and structure beyond that available from conventional angiography [1].

The limitations of conventional coronary angiography are increasingly recognized even though it remains the cornerstone of coronary imaging and the standard technique for guiding percutaneous coronary intervention (PCI). As a luminographic technique, a two-dimensional representation of the coronary lumen is provided by the angiography but also offers limited information about plaque burden, plaque composition, vessel wall architecture, and high-risk features such as thin-cap fibroatheromas, calcification, and lipid-rich cores. Many of these limitations are addressed by intravascular ultrasound (IVUS), which provides cross-sectional images of the coronary vessel wall. It also assesses the plaque morphology, vascular remodeling, external elastic membrane boundaries, and lumen dimensions. However, interpretation of conventional IVUS remains highly dependent on the operator and time-consuming. It is also subject to inter- and intra-observer variability in concluding the findings, particularly when quantitative analysis is required for extensive pullback sequences. Artificial intelligence (AI) is a broad term that encompasses computer algorithms capable of performing tasks that are usually done by humans. Within this field, one particular type has been developed that is called “machine learning (ML).” ML is a form of AI in which the algorithms can learn and adapt without being programmed to do so explicitly. One of the most common forms of ML is “deep learning (DL),” which is used primarily to perform image segmentation or classification.

AI has proven successful in a number of IVUS interpretation tasks relevant to procedural planning. Automatically determining the degree of vessel attenuation and calcification has proved to be accurate in the context of expert diagnosis [1]. Deep-learning methods have also been used to automatically identify lumen, vessel, and stent borders [2], and lumen, plaque, and calcified regions [3], streamlining a time-consuming process for analysts. The ability to segment images has also been harnessed in procedural planning, where automated vessel and lumen measurements are used for PCI, including guidance in balloon and stent sizing, thus reducing operator reliance on manual caliper-based measurement [4].

Further segmentation strategies have improved the accuracy of lumen and external elastic membrane delineation with considerable reduction in analysis time versus manual tracing [5]. In addition to anatomical segmentations, AI technologies have also been applied to plaque and tissue characterization: integrated backscatter IVUS with DL allows detection of lipid-rich, vulnerable plaque and calculation of calcified plaque content [6], whereas alternative gradient-boosting strategies have enabled automated lumen segmentation with good agreement against manual measurements [7]. Recently, combined DL frameworks that simultaneously segment the lumen, media-adventitia border, and calcified plaque have emerged, consolidating these previously separate interpretive tasks into a single interpretive model with potential to streamline the overall interpretation workflow in the catheterization laboratory [8].

The clinical relevance of these developments is closely related to the established role of IVUS in PCI optimization. IVUS-guided procedures can support more effective stent deployment than angiography alone by providing information about lesion length, vessel dimensions, stent expansion, and plaque burden [9]. Clinical studies have reported that IVUS-guided PCI is associated with lower rates of restenosis, improved stent expansion, lower rates of stent thrombosis, and reduction in major adverse cardiovascular events, particularly in patients with high-risk coronary anatomy and complex lesions [10]. As the use of IVUS increases in contemporary catheterization laboratories, the volume of imaging data generated during routine procedures is also growing. This has enabled the need for automated image-analysis tools that can maintain reproducibility and accuracy while efficiently fitting into clinical workflows. AI-assisted IVUS interpretation may improve consistency in diagnosis while reducing the time and expertise required for comprehensive image analysis, which has been indicated by recent evidence [11-13].

Beyond procedures, AI-enhanced IVUS may also improve the assessment of coronary atherosclerosis over time. Serial evaluation of plaque progression and regression during medical therapy has been enabled by automated quantitative analysis, providing a more objective means of monitoring disease evolution and treatment responses [14,15]. Reviews of AI in intracoronary imaging have emphasized its potential in improving segmentation, reducing observer variability, automating plaque characterization, and streamlining clinical workflows [16]. Expert consensus documents have also acknowledged the increasing role of computational approaches for intravascular imaging while also noting the need for standardized validation prior to the widespread adoption [17]. As such, AI may play an important role in the future of precision cardiovascular medicine, particularly when used in combination with standard Intracoronary imaging such as IVUS [18]. Multimodal machine-learning methods that use IVUS with optical coherence tomography and coronary computed tomography angiography may enhance the assessment of coronary plaques and support individualized cardiovascular risk assessment [19].

Regardless of these advances, there are still challenges to be addressed before AI-assisted IVUS analysis can be fully integrated into routine clinical practice. Existing studies vary substantially in study design, imaging platforms, AI architectures, patient populations, outcome measures, validation methods, and reporting standards. This heterogeneity makes it difficult to compare findings across studies and limits the generalizability of the available evidence. There has been an increasing number of studies in recent years that have focused on both the clinical potential for AI for coronary plaque characterization and evidence gaps that continue to be difficult for broader implementation [20]. These limitations include the need for rigorous external validation, standardized reporting frameworks, and prospective multicenter clinical trials. Therefore, this systematic review aims to synthesize the current evidence on the use of AI for the automated analysis and interpretation of IVUS in CAD. Particularly, attention is drawn to advances in automated segmentation, plaque characterization, quantitative image analysis, and diagnostic accuracy, as well as identifying potential limitations, gaps in evidence, and future perspectives for incorporating AI-assisted IVUS into routine cardiovascular practice.

## Review

Methodology

Study Design

This systematic review has been performed according to the Preferred Reporting Items for Systematic Reviews and Meta-Analyses (PRISMA) 2020 statement [21]. The review protocol has been prospectively registered in the International Prospective Register of systematic Reviews (PROSPERO; registration number CRD420261454806) before the start of the study selection process.

Types of Studies Included

Original peer-reviewed research articles, including prospective or retrospective observational studies, diagnostic accuracy studies, and clinical or technical validation studies involving human coronary IVUS datasets, were considered eligible.

Inclusion Criteria

Studies on human coronary IVUS and CAD; studies evaluating AI, ML, or DL algorithms for analyzing or interpreting coronary IVUS images for purposes such as lumen segmentation, vessel border detection, plaque characterization, tissue classification, calcium detection, lesion identification, or quantitative vessel measurements; and full-text articles published in the English language were included.

Exclusion Criteria

Reviews, systematics reviews, meta-analyses, editorials, letters, and studies protocols; abstracts presented at conferences without a full published article; animal or in vitro studies; studies reporting on non-coronary IVUS (e.g., peripheral or carotid); studies in which IVUS was used as a reference or comparator standard against which other imaging or analytical methods were compared; case reports or series; and duplicates were excluded.

Information Sources

A comprehensive search strategy was used to identify relevant studies in PubMed and Cochrane CENTRAL. The databases were searched from inception to July 23, 2026, for PubMed and from inception to July 26, 2026, for Cochrane CENTRAL, with no restrictions on publication date. In addition, reference lists of included studies and reviews were hand-searched to identify further eligible studies that might have been missed by the initial search strategy.

Search Strategy

The search strategy comprised combinations of medical subject headings and free-text words related to three concept blocks: (i) “artificial intelligence”, “machine learning”, “deep learning”; (ii) “intravascular ultrasound”; and (iii) “coronary artery disease” using the Boolean operators AND/OR. The search was limited to human studies published in English (Table 1).

**Table 1:** Search strategy and databases The table shows a summary of databases searched, Boolean search strategy, filters applied, and number of records retrieved (Total number of studies, n=802).

Database	Keywords / MeSH Terms Used (summary)	Filters Applied	Results Retrieved	Date Searched
PubMed	("Artificial Intelligence"[Mesh] OR "Machine Learning"[Mesh] OR "Deep Learning"[Mesh] OR AI OR CNN OR "Neural Network*" OR segmentation OR classification OR "plaque characterization") AND ("Intravascular Ultrasound"[Mesh] OR IVUS) AND ("Coronary Artery Disease"[Mesh] OR CAD OR coronary OR PCI OR stent OR plaque)	Humans; English language	216	23/07/2026
Cochrane CENTRAL	(Artificial Intelligence OR AI OR Machine Learning OR Deep Learning OR Neural Network* OR CNN OR segmentation OR classification OR Plaque Characterization) AND (Intravascular Ultrasound OR IVUS) AND (Coronary OR Coronary Artery Disease OR CAD OR PCI OR plaque OR stent)	Humans; English language	586	26/07/2026

Population, Intervention, Comparison, and Outcome (PICO) Framework

Table 2 summarizes the Population, Intervention, Comparison, and Outcome (PICO) framework used to guide study selection and data extraction for this systematic review.

**Table 2:** PICO framework CAD, coronary artery disease; IVUS, intravascular ultrasound; AI, artificial intelligence; ML, machine learning; DL, deep learning; ROC, receiver operating characteristic; PICO, Population, Intervention, Comparison, and Outcome

Component	Description
Population (P)	Adults with CAD undergoing coronary IVUS during percutaneous coronary intervention.
Intervention (I)	AI, ML, or DL algorithms used for automated analysis or interpretation of coronary IVUS images (e.g., lumen/vessel segmentation, plaque characterization, calcium detection, stent assessment).
Comparator (C)	Conventional/manual IVUS interpretation by expert clinicians, semi-automated software, or validated reference standards.
Outcomes (O)	Diagnostic accuracy, segmentation performance (Dice similarity coefficient, IoU), sensitivity, specificity, accuracy, area under the ROC curve, plaque characterization accuracy, vessel/lumen measurement accuracy, agreement with expert interpretation, and processing time.

Study Selection and Data Synthesis

The records retrieved from the databases (PubMed and Cochrane CENTRAL) were imported into Rayyan (Rayyan Systems Inc., Cambridge, MA, USA) for screening and deduplication. Following the removal of duplicates, titles and abstracts were screened against the inclusion-exclusion criteria. The reports that met the initial screening criteria were selected for full-text eligibility screening. The documents that failed the full-text eligibility screening were excluded, with reasons for exclusion recorded (including review articles or editorials, studies involving non-coronary IVUS, animal or in vitro studies, and studies involving IVUS with non-AI-based analysis). The reports that passed the eligibility screening were included in the subsequent stages of the study. The PRISMA 2020 flow diagram in Figure 1 indicates the number of screened, excluded, and included records in each stage of this scoping review.

**Figure 1:**
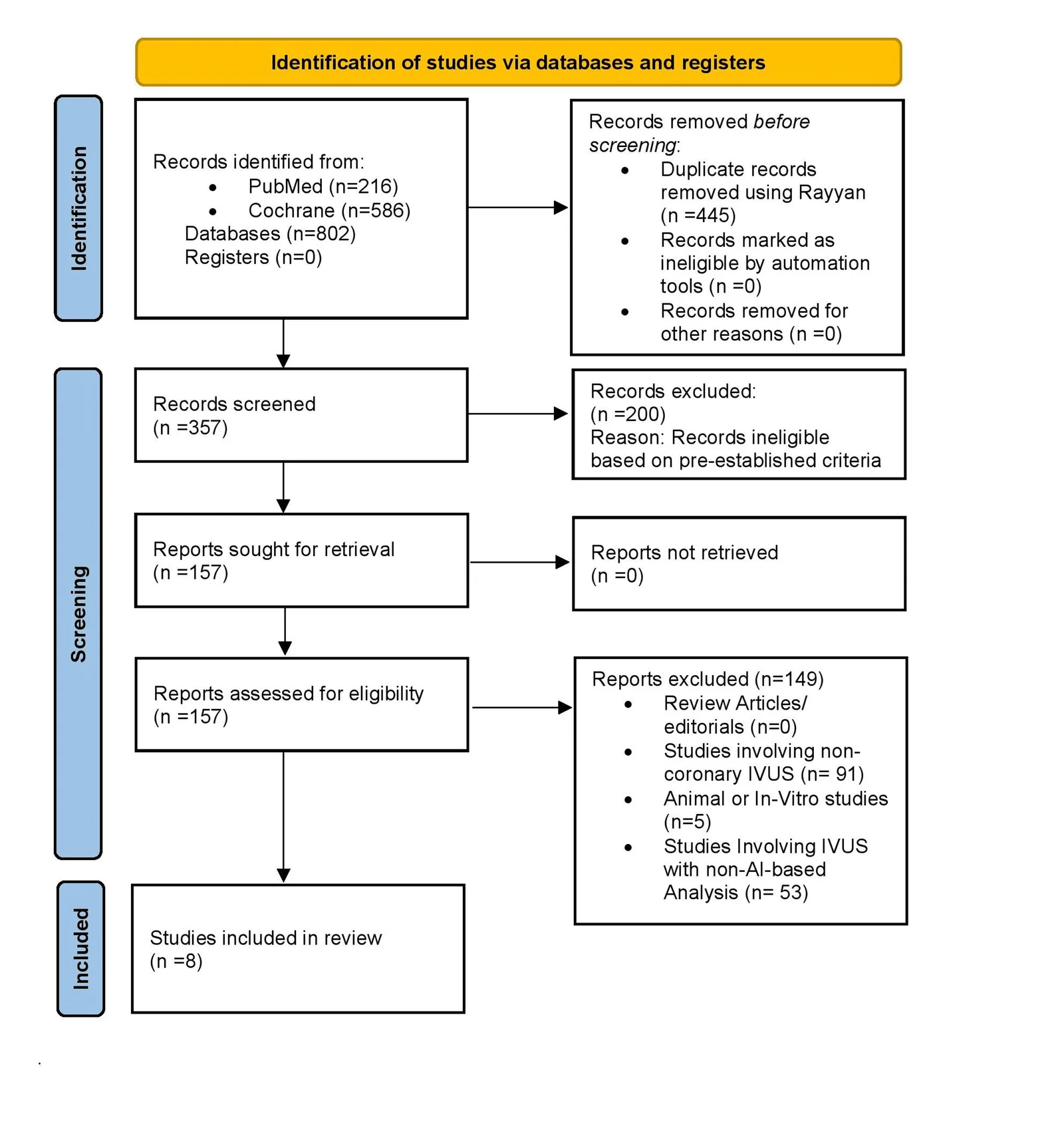
PRISMA 2020 flow diagram PRISMA, Preferred Reporting Items for Systematic Reviews and Meta-Analyses; IVUS, intravascular ultrasound

Two independent reviewers screened every record’s title and abstract to identify documents that met the eligibility criteria. However, discrepancies that arose during the screening of titles and abstracts were resolved through discussion with a third reviewer. A similar step was undertaken when screening the full text of the records against the eligibility criteria.

Data Extraction

Two independent reviewers extracted the data from each study using an extraction form, which was pilot-tested. The following variables were extracted: author, year of publication, country, study design, AI/model or architecture used, application of IVUS, dataset and sample size, reference standard, and diagnostic performance (sensitivity, specificity, accuracy, area under the receiver operating characteristic curve, Dice similarity coefficient (DSC), intersection over union (IoU), and correlation coefficient, when reported). Disagreements between the reviewers were resolved through discussions and consensus.

Risk-of-Bias Assessment

The methodological quality of the included studies was assessed by two reviewers using the Quality Assessment of Diagnostic Accuracy Studies-2 (QUADAS-2) tool [22], which assesses the risk of bias of a study across four domains: patient selection, index test, reference standard, and flow and timing. Any disagreement between the reviewers was resolved by discussion. A summary of risk-of-bias judgments across domains and studies, together with graphic displays, was generated using the robvis tool. Specifically, a traffic-light plot and a bar plot were produced.

Certainty of Evidence Assessment

The overall certainty of evidence for the included studies was assessed using the Grading of Recommendations Assessment, Development, and Evaluation (GRADE) approach. The GRADE methodology considers risk of bias, inconsistency, indirectness, imprecision, and publication bias as domains that modify the certainty of evidence across the body of evidence. Certainty was classified as high, moderate, low, or very low.

Data Synthesis

The included studies were grouped according to the AI model architecture, IVUS application, reference standard, and diagnostic performance.

Results

Study Selection

The database search identified a total of 802 records (PubMed, n=216; Cochrane Central, n=586). After removing 445 duplicates in Rayyan, a total of 357 records were obtained for title and abstract screening. Of these, 200 records were excluded due to pre-established inclusion and exclusion criteria, leaving 157 reports searched for full-text availability. All 157 studies were successfully retrieved (0 not retrieved). After the full-text assessment, 149 reports were excluded, such as review articles or editorials, studies with non-coronary IVUS, animal or in vitro studies, and studies with IVUS that do not involve AI-based analysis. In total, eight studies met all the inclusion criteria and were included in the systematic review (Figure 1).

Study Characteristics

A total of eight studies published from 2020 to 2025 were included in the study. The studies were carried out in South Korea, the United States, Japan, Taiwan, and China. One of the included studies reported an analysis that involved a multicenter multinational sample of patients in Asia, Europe, and the United States. All the selected studies had a retrospective diagnostic accuracy or technical validation design. The number of images per study ranged from 713 (18 patients) to more than 45,000 (over 1,500 IVUS runs). The following AI-based algorithms were applied: convolutional neural networks, U-Net and U-Net++, Efficient-UNet, DeepLabv3-based models, and gradient boosting methods. The included studies reported the application of IVUS in a wide range of research areas such as lumen, vessel and stent segmentation, attenuation and calcification quantification, plaque and tissue characterization, and PCI-guided stent/balloon sizing (Table 3).

**Table 3:** Characteristics of included studies assessing AI for automated analysis of coronary IVUS Reference standards across the included studies were primarily based on manual expert annotation or segmentation by expert cardiologists or core-laboratory analysts, with two studies (Masuda et al. [6] and Cui et al. [7]) utilizing an imaging-derived surrogate reference (integrated backscatter IVUS and manually measured lumen area, respectively) instead of complete pixel-level expert segmentation. AI, artificial intelligence; CNN, convolutional neural network; IVUS, intravascular ultrasound; ML, machine learning; MICCAI, Medical Image Computing and Computer-Assisted Intervention; LOSO–CV, leave-one-subject-out cross-validation

Study, Year	Country	Design	Sample / Dataset	AI Model	Validation
Cho et al., 2021 [1]	South Korea	Retrospective AI study	598 arteries; 137,989 frames	CNN (EfficientNet)	Internal
Nishi et al., 2021 [2]	USA (+ external cohort, Japan)	Retrospective AI study	1,576 IVUS runs; 45,449 images	Deep learning (DeepLabv3)	Internal and external
Masuda et al., 2025 [6]	Japan	Retrospective AI study	1,098 training / 1,100 validation images	7-layer U-Net++	Internal
Jeong et al., 2024 [5]	South Korea	Retrospective AI study	1,063 patients + 111 pullbacks	Efficient-UNet	Independent
Shinohara et al., 2021 [3]	Japan	Retrospective AI study	3,738 images; 24 patients	U-Net	Internal
Matsumura et al., 2023 [4]	Multinational	Retrospective ML study	8,076 training; 437 test images	Machine learning (U-Net)	Independent
Cui et al., 2020 [7]	China (+ Singapore data)	Methodological study	MICCAI dataset + 3 real-patient sets	Gradient boosting	External
Li et al., 2021 [8]	Taiwan	Deep learning study	713 images; 18 patients	Deep CNN (cascaded U-Nets)	Internal (LOSO-CV)

Diagnostic Performance Outcomes

All studies included in this review reported diagnostic or segmentation performance outcome measures for AI-based analysis of coronary IVUS. Reported segmentation performance varied by metric. Reported segmentation performance varied between the different metrics, with values expressed as the DSC ranging from 0.67 to 0.97 across structures, and values expressed as the intersection over union (IoU) ranging from 0.66 to 0.97 across studies and structures. For both metrics, the highest agreement was generally seen for lumen and vessel or EEM segmentation, with relatively lower agreement for calcification and stent segmentation. Where classification outcome measures were reported, sensitivity ranged from 62% to 90% and specificity from 88% to 99% for attenuation, calcification, and lipid-rich plaque detection tasks across studies. Those studies that evaluated quantitative agreement with expert measurement (e.g., lumen/vessel area, balloon sizing) reported correlation coefficients generally above r = 0.90 (except vessel-area agreement in images with poor vessel border visibility). Several studies also reported considerable reductions in analysis time versus manual expert measurement, most notably Jeong et al. [5], who reported a reduction from 187.5 minutes (manual) to 13.2 seconds (AI-based) for segmentation of a 20-mm lesion segment (Table 4).

**Table 4:** Diagnostic performance outcomes of artificial intelligence applications for automated IVUS analysis Disaggregated by metric type: DSC-reporting studies ranged from 0.67 to 0.97; IoU-reporting studies (including Jaccard similarity) ranged from 0.66 to 0.97. IoU, intersection over union (Jaccard similarity is mathematically equivalent to IoU); AUC, area under the receiver operating characteristic curve; DSC, Dice similarity coefficient; EEM, external elastic membrane; AI, artificial intelligence; PCI, percutaneous coronary intervention; AP, average precision; IVUS, intravascular ultrasound

Study	Primary Task	Metric Type	Key Diagnostic Performance Metrics
Cho et al., 2021 [1]	Attenuation and calcification quantification	Accuracy / sensitivity–specificity	Angle-level accuracy 98%; attenuation sensitivity 80% / specificity 96%; calcification sensitivity 86% / specificity 97%; burden-index correlation r = 0.89–0.95
Nishi et al., 2021 [2]	Lumen, vessel, and stent segmentation	IoU	Mean IoU 0.80 ± 0.20; correlation 0.98 (lumen), 0.96 (vessel), 0.96 (stent); external validation correlation 0.99
Masuda et al., 2025 [6]	Plaque segmentation and tissue characterization	AUC / correlation	%lipid area R = 0.90; lipid-rich plaque detection AUC 0.919, sensitivity 62%, specificity 94%
Jeong et al., 2024 [5]	Lumen and EEM segmentation	DSC	DSC 0.93 (lumen) / 0.97 (EEM); minimal lumen area correlation r = 0.92–0.93; 20-mm segment: 13.2 s (AI) vs 187.5 min (manual)
Shinohara et al., 2021 [3]	Lumen, plaque, calcification, and stent segmentation	IoU / accuracy	Narrowed-lumen accuracy 0.97; severe-calcification accuracy 0.98; mean IoU 0.66; stent recall only 0.64
Matsumura et al., 2023 [4]	PCI planning: segmentation and balloon sizing	Correlation / agreement	Lumen correlation r = 0.991; vessel r = 0.967; balloon-size agreement 92.4% (vessel + lumen diameter)
Cui et al., 2020 [7]	Lumen segmentation	IoU (Jaccard)	Public dataset Jaccard similarity 96.8% (= IoU 0.968); real-patient percent error 1.67–1.97%; regression R = 0.969
Li et al., 2021 [8]	Plaque, lumen, media, and calcification detection	DSC	Media-adventitia / lumen DSC > 0.90; calcification detection AP = 0.73, DSC = 0.67 (focal loss)

Performance was consistently found to be lower for stent-related tasks in comparison to lumen or vessel segmentation. Shinohara et al. reported only a class level IOU of 0.05 for stent segmentation despite high accuracy for lumen-and calcification-related classification, reflecting the relatively small and variable appearance of stent struts within IVUS frames [3]. Similarly, Nishi et al. and Matsumura et al., who specifically evaluated stent and balloon-sizing applications relevant to PCI guidance, reported stronger agreement (correlation coefficients of 0.96-0.98 and agreement rates up to 97.0%, respectively), reflecting the utility of models purpose-built and trained on larger, stent-specific datasets [2,4].

Risk-of-Bias Assessment

Risk of bias was assessed using the QUADAS-2 tool in four domains: patient selection, reference standard, index test, and flow and timing. Overall, satisfactory methodological quality of included studies was obtained with no study rated at high risk of bias in any domain. Risk of bias was most frequently reported in the patient selection domain, where five (62.5%) of the eight studies were rated as raising some concerns, mainly due to the retrospective, single-center design with exclusion of poor-quality or artifact-containing images or due to demographic differences between training and validation cohorts. The index test domain was rated as low risk across all eight studies, reflecting consistent assessment of AI models on either independent or held-out test datasets. The reference standard domain raised some concerns in three (37.5%) of the eight studies, relating to the use of imaging-derived surrogate reference standards (integrated backscatter IVUS, manually measured lumen area) or single-annotator initial segmentation, rather than fully independent or histological gold standard. The flow and timing domain was rated as low risk across all eight studies. Two studies (Matsumura et al. [4] and Jeong et al. [5]) were rated as being at overall low risk of bias across all four domains. The distribution of risk of bias across studies is illustrated in Figures 2, 3, while supporting rationale for each judgment is presented in Table 5.

**Figure 2:**
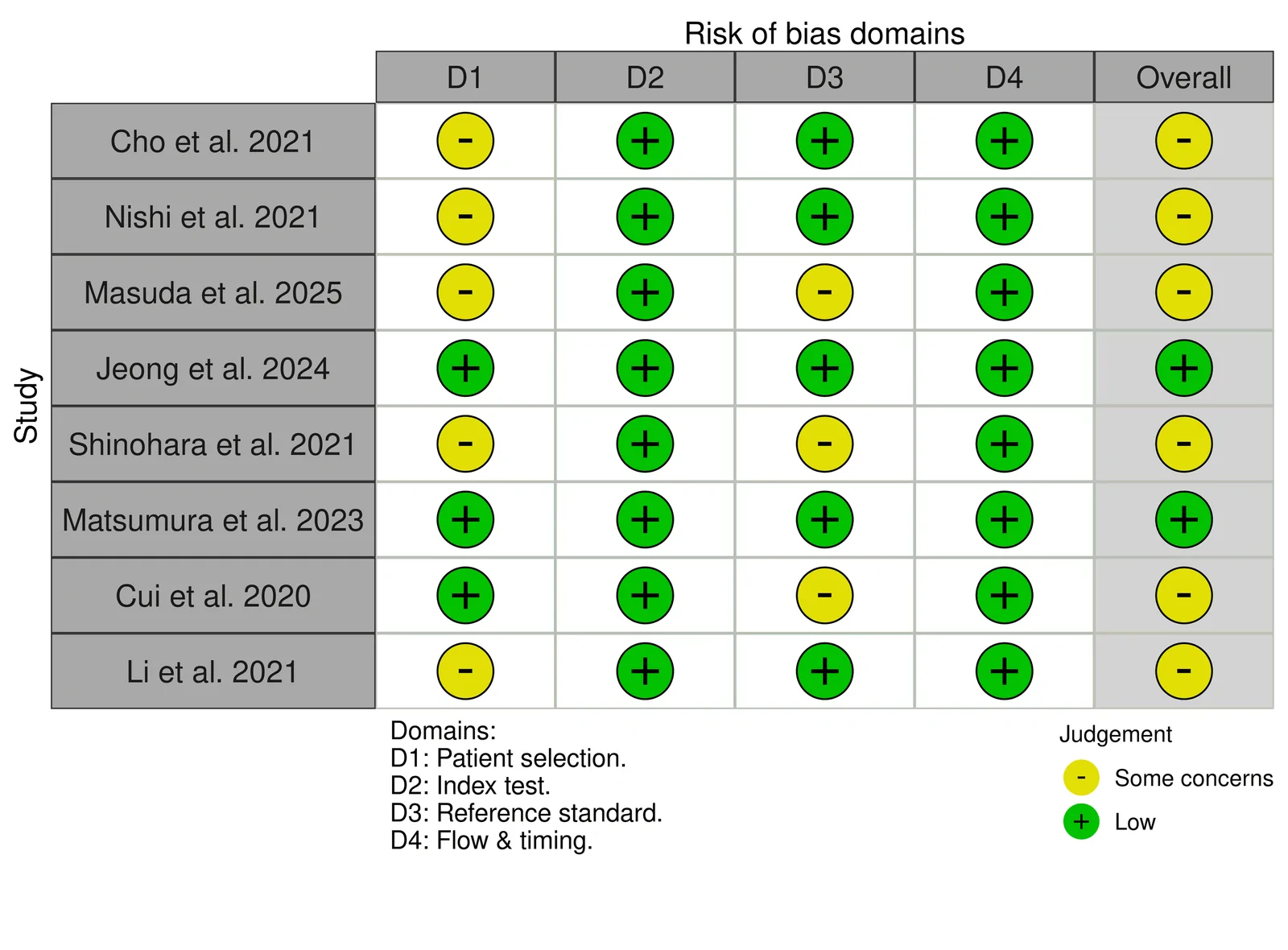
Traffic light plot of QUADAS-2 risk-of-bias judgments across the included studies Studies: Cho et al. [1], Nishi et al. [2], Masuda et al. [6], Jeong et al. [5], Shinohara et al. [3], Matsumura et al. [4], Cui et al. [7], Li et al. [8] QUADAS-2, Quality Assessment of Diagnostic Accuracy Studies-2

**Figure 3:**
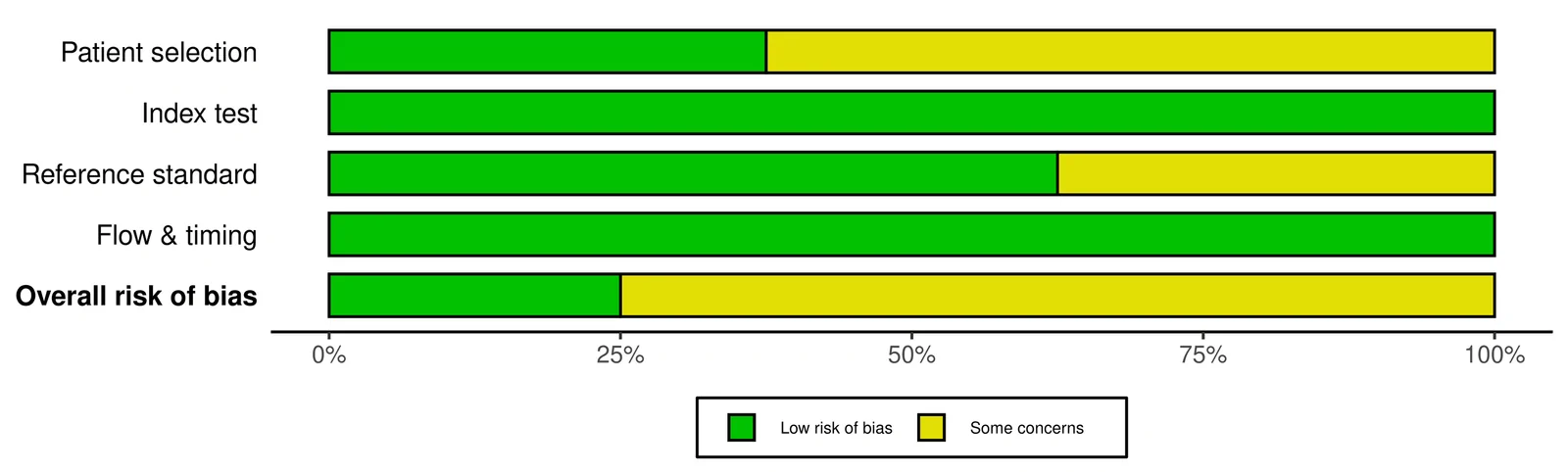
Summary plot of QUADAS-2 risk-of-bias judgments across domains (total number of studies n=8) QUADAS-2, Quality Assessment of Diagnostic Accuracy Studies-2

**Table 5:** Summary of QUADAS-2 risk-of-bias judgments across domains DL, deep learning; IVUS, intravascular ultrasound; BMI, body mass index; ACS, acute coronary syndrome; IB-IVUS, integrated backscatter intravascular ultrasound; EEM, external elastic membrane; MICCAI, Medical Image Computing and Computer-Assisted Intervention; QUADAS-2, Quality Assessment of Diagnostic Accuracy Studies-2

Study	Patient Selection	Index Test	Reference Standard	Flow and Timing
Cho et al., 2021 [1]	Some concerns: retrospective cohort with exclusion of stented lesions and poor-quality images	Low: degree-wise classifier evaluated on a fully independent, non-overlapping test set	Low: ground truth labelled by two blinded expert observers with third-reviewer adjudication	Low: consistent 0.4-mm interval sampling with complete case accounting
Nishi et al., 2021 [2]	Some concerns: retrospective single-center cohort; images with large branches or extensive artefact excluded before analysis	Low: DL model evaluated on an independent test dataset and an external 60-MHz validation cohort	Low: manual expert annotation performed using validated quantitative IVUS software	Low: defined training/validation/test split with complete case inclusion
Masuda et al., 2025 [6]	Some concerns: training and validation cohorts differed significantly in BMI and ACS prevalence	Low: 7-layer U-Net++ segmentation evaluated against an independent validation cohort	Some concerns: IB-IVUS used as a signal-based surrogate reference rather than a histological gold standard	Low: consistent 1-mm interval sampling with defined case inclusion
Jeong et al., 2024 [5]	Low: random patient-level allocation to training/test sets plus an independent temporal validation cohort	Low: efficient-UNet model evaluated blinded to expert measurements on held-out data	Low: manual lumen/EEM tracing performed by experienced core-laboratory analysts	Low: consistent 0.2-mm interval sampling with complete case inclusion
Shinohara et al., 2021 [3]	Some concerns: small single-center retrospective cohort (24 patients) after excluding pre-dilated or poor-quality cases	Low: U-Net model evaluated on a randomly selected, held-out test set of images	Some concerns: manual pixel-level segmentation performed initially by a single annotator, refined by consensus	Low: fixed 0.5-mm interval sampling with all extracted frames accounted for
Matsumura et al., 2023 [4]	Low: pre-specified, geographically diverse training and fully independent test cohorts	Low: U-Net segmentation assessed against expert analysis on an independent test set	Low: expert analysis performed using standardized simulator software as the reference	Low: consistent slice- and lesion-level analysis with complete reporting
Cui et al., 2020 [7]	Low: public MICCAI challenge dataset supplemented with real-patient pullback data	Low: gradient boosting model trained and evaluated on a designated public test partition	Some concerns: real-patient validation relied on manually measured lumen area rather than full pixel-level ground truth	Low: consistent frame sampling with complete case inclusion within each dataset
Li et al., 2021 [8]	Some concerns: small single-center cohort (18 patients) with incompletely reported demographic data	Low: cascaded U-Net model evaluated using leave-one-subject-out cross-validation	Low: manual annotation performed by two cardiologists blinded to clinical information	Low: all extracted frames accounted for across cross-validation folds

​​​*Certainty of Evidence*

Quality of evidence was examined in the domains of risk of bias, inconsistency, indirectness, imprecision, and publication bias, and classified into high, moderate, low, or very low certainty evidence using the GRADE framework [23]. Serious concerns about risk of bias were identified, reflecting the QUADAS-2 findings that there were some concerns in five of the eight included studies for the patient selection domain and three for the reference standard domain. Serious inconsistency was also identified, reflecting substantial variability in the AI tasks assessed (segmentation, classification, and quantitative measurement), reference standards, and outcome measures across the included studies, which also precluded quantitative meta-analysis. No serious concerns were identified regarding indirectness, as all included studies directly evaluated AI-based analysis of coronary IVUS against an accepted reference standard in patients with CAD, consistent with the review question. Imprecision was a possible concern in a minority of the studies of small sample size (18-24 patients). Publication bias could not be formally assessed but is a potential limitation as studies reporting favorable AI performance may be more likely to be published. Taking all domains together, the overall certainty of evidence of the diagnostic performance of AI in automated coronary IVUS analysis was rated as low.

Summary of Findings

AI algorithms, including convolutional neural networks, U-Net-based architectures, and gradient boosting methods, showed consistently high performance on automated segmentation and characterization of coronary IVUS images, with most consistent results for lumen and vessel segmentation and less consistent performance for stent detection and calcification characterization. Methodological quality of included studies was generally satisfactory, but risk of bias in terms of patient selection and reference standard was reported in more than half of the studies. Due to heterogeneity in AI tasks, reference standards, and outcome measures between included studies, a quantitative meta-analysis was not appropriate; findings are therefore reported as a qualitative, narrative synthesis.

Discussion

Diagnostic Performance of AI in Automated IVUS Interpretation

Taken together, the eight studies in this review frame a consistent picture: AI performs very well at the geometric tasks, which form the backbone of IVUS interpretation, specifically lumen and vessel segmentation [1-8]. Each of the included papers was able to show that their model can trace vascular boundaries, estimate plaque burden, and characterize tissue composition often with a fraction of the time required for manual analysis. The clinical impact of such methods has been shown to be significant, as IVUS-guided PCI improves outcomes, though it remains underused in everyday practice, possibly due to the time-consuming nature of the analysis and the need for specialized expertise [2,6].

Several of these studies report particularly impressive results, with the most notable being the one by Jeong et al. [5], where the Efficient-UNet model achieved a DSC of 0.93 for the lumen and 0.97 for the external elastic membrane, with minimal lumen area closely tracking the expert’s results (r = 0.92-0.93). Another paper that reported results of a similar level was the one by Nishi et al. [2], who used a variation of the DeepLabv3 architecture. While their results were slightly lower (DSC 0.93 for the lumen and 0.91 for the external elastic membrane), they were obtained on a much larger set of 45,449 images from 1,576 pullbacks and also demonstrated the validity of their claims on an external test set of 60-MHz acquisitions. Matsumura et al. [4] reported very similar results in terms of the DSC (lumen: 0.991; vessel area: 0.967) and showed an improvement in balloon sizing ranging from 70.6% to 92.4% when using lumen diameter in conjunction with vessel diameter.

The same cannot be said of all the studies; however, Shinohara et al. [3] serves as an example of the model that performed reasonably well on the classification task (narrowed lumen: 0.97; severe calcification: 0.98) but significantly worse on the segmentation (mean IoU: 0.66), with the latter being largely detected by the poor performance of the stents (IoU: 0.05). This serves to remind that classification and segmentation are two different tasks and that a model’s performance on one does not necessarily translate to the other.

Comparative Analysis Across AI Architectures and Tasks

Read in publication order, these eight studies also represent a rough chronological progression of how AI approaches to IVUS have developed. Cui et al. did not involve a deep neural network; instead, they used a gradient boosting approach that achieved a Jaccard similarity score of 96.8% on publicly available challenge data and a percent error of less than 2% on the lumen area of real-patient data. Notably, this validation was performed against manual lumen area measurements [7]. Subsequently, research shifted toward U-Net-based architectures. Li et al. [8] developed a three-stage cascaded U-Net to specifically identify calcified plaque from surrounding tissue, achieving an average precision of 0.73 when a focal loss function was employed.

The later studies continue the trend. Masuda et al. [6] took a slightly different approach by using a 7-layer U-Net++ to generate a “virtual” integrated backscatter image from grayscale IVUS, which correlated well with genuine IB-IVUS tissue composition (R = 0.81-0.90) and yielded an AUC of 0.919 for identifying lipid-rich plaque. Jeong et al. [5] and Matsumura et al. [4], the two most recent papers in this review, both used larger and, in the latter’s case, higher-definition datasets and both achieved the closest agreement to expert measurement of any of the included studies. The progression is not definitive evidence that newer always equals better, but it does fit a pattern seen in this literature: bigger, better-curated training sets tend to deliver genuine performance improvements, sometimes more than tweaks to the network architecture.

Stent detection is perhaps the area where this principle is best illustrated. Shinohara et al.’s [3] poor performance on stents was not an architecture issue per se, but a combination of stents making up only 12.3% of their training images and the strong similarity between stent struts and calcified plaque on grayscale IVUS. Nishi et al. [2] and Matsumura et al. [4], who both used considerably larger and more stent-laden datasets, had no such issues, reporting stent correlations of 0.96-0.98 and an acute stent area agreement of 97.0%. The takeaway appears to be that the choice of network is less important than ensuring that the model has seen enough examples of the target class to learn its characteristics.

Sources of Heterogeneity

It is worth noting that these eight studies differ in many respects, which is the primary reason why a meta-analysis was not attempted. Notably, the model architecture, the size of the dataset, the IVUS frequency, the reference standard, and the outcome metric all differed considerably [1-8]. Indeed, the number of images ranged from 713 images across 18 patients in Li et al.’s study [8] to 45,449 images from 1576 runs in Nishi et al.’s study [2], and in general the larger, multi-institutional studies yielded better-performing models than the smaller single-center studies [2,4,6].

Most of these studies also employed varying reference standards. Although the majority of these studies used manual expert annotation as a reference standard [1,2,4,5,8], others used integrated backscatter IVUS - a signal processing surrogate rather than a histological standard - to evaluate their results [3], while still others evaluated their performance on patient data by comparing the manual luminal area measurement to their segmentation result only [7]. This heterogeneity in the reference standard precluded a direct comparison of the agreement statistics between these studies. When combined with the fact that the earlier studies [1,5,7,8] used 20-45 MHz transducers, whereas the later [4,6] used 60 MHz high-definition systems, this makes it difficult to compare the actual input image characteristics between the different studies.

Finally, the outcome metrics reported by these studies were not always directly comparable to one another. Specifically, while some studies reported DSC or IoU [2,4,5,7,8], others reported sensitivity, specificity, or AUC [1,3,5], and some simply reported the correlation coefficient or percentage agreement [2,6,7]. As such, a calculation of a pooled effect estimate would not have been informative, as it would have resulted in a value that is difficult to interpret. A narrative synthesis, while perhaps less statistically satisfying, was the more honest option given what these studies actually measured.

Clinical Utility in Optimizing PCI

The most promising aspect of this review’s findings is the implication it holds for the time. Jeong et al. found that AI-based segmentation of a 20-mm lesion took 13.2 seconds versus 187.5 minutes of manual work by an expert [5]. Nishi et al. found an equally impressive number: approximately 4 seconds for every 100 images by AI versus 30 hours for an expert [2]. If these kinds of results can be reproduced outside the research setting, IVUS with AI-assistance has the potential to be practically applied in real time within the catheterization lab without significantly increasing the duration of procedure.

It should be noted that AI could potentially aid not only in measurement but also in the decision-making process. Matsumura et al. found that the use of lumen diameter in addition to vessel diameter increased the percentage of correctly sized balloon selection from 70.6% to 92.4% [4]. This is immediately applicable information to have during a PCI. In a similar vein, Masuda et al. and Cho et al. found that AI was able to perform plaque and tissue characterization, identifying lipid-rich plaque or vulnerable plaque and quantifying the calcification burden, respectively, with both being known contributory factors to complications [1,6]. While none of these results can replace the skill and experience of a human physician, it is evident that AI has the potential not only to measure but also to stratify risk, providing information applicable to a PCI.

With that being said, the findings of Shinohara et al. regarding AI and stents and must be considered [3]. A system that can segment lumen and vessel, but has difficulty with stents, cannot be used in isolation for a PCI. It has the potential to be a helpful tool, but at this time, it cannot make decisions about stents on its own. It must be combined with the expertise of an interventional cardiologist to achieve the best results.

Strengths and limitations

The review has certain methodological strengths, including adherence to the PRISMA 2020 guidelines, registration in the PROSPERO database, a pre-specified search strategy in two databases, and independent study selection and data extraction by two reviewers to minimize bias. The risk of bias was assessed using the QUADAS-2 tool and visualized using Robvis.

However, several limitations were noted in the review, including the fact that all eight studies were retrospective, which limits the generalizability of findings to pragmatic, prospective studies. The meta-analysis was not performed because the pooling of results would not be meaningful due to the heterogeneity of studies investigating different aspects of diagnostic performance (segmentation, classification, level of agreement), varying reference standards, and different outcome measures used in each of the studies. The search strategy was limited to only two databases (PubMed/MEDLINE and Cochrane CENTRAL). For AI and biomedical imaging topics, Embase and Scopus are important sources given their strong coverage of engineering, computer science, and biomedical technology journals; their exclusion may have led to missed relevant studies and represents a notable limitation of this review. Additionally, the search was last performed on July 23, 2026, and July 26, 2026, and no further database alerts or manual searches were carried out before submission of the manuscript; thus, any eligible studies published after this date are not included in the present review. Finally, the five [1,2,3,5,8] out of eight studies were limited by the selection of patients, as single-center retrospective studies were more likely to exclude poor-quality scans or those presenting with artifacts, thus affecting the generalizability of findings to clinical practice. Lastly, publication bias cannot be ruled out, as studies with positive AI results are more likely to be published.

Future directions

The obvious next step for validation is the prospective multicenter study - a stage that has been largely neglected in the current literature [1,3-5,7,8]. Equally importantly, the field would profit from establishing a consensus on the way performance is reported: the current mixture of Dice scores, IoU values, correlation coefficients, and accuracy quotients contributes to the overall confusion of the field. Considering the consistent relative difficulties in achieving satisfactory stent segmentation [2,5,6], the former direction, as well as increased emphasis on creating larger and more balanced stent-containing training sets, should be prioritized in future model development efforts.

Finally, the evaluation paradigm could be adjusted to focus more on the clinically relevant aspects of the technology, such as its ability to affect the procedural time, complication rates, and late cardiovascular outcomes, rather than the segmentation accuracy per se. Furthermore, given the lack of external validity in the current state of the art, only two out of eight studies [2,3] reported having tested their models on data originating from sources different from the ones used in training; there is a need to engage in a more thorough validation before any of these models could see a clinical application in the real world with its multitude of IVUS scanners, transducer frequencies, and patient acoustical profiles.

In addition to the performance characteristics of the models, practical considerations regarding clinical translation of AI analysis tools should be addressed. Notably, the majority of the algorithms included in this review were developed and validated offline, and it is unclear whether such applications could be incorporated into existing commercial IVUS consoles or catheterization laboratory workstations for real-time processing. Variations in imaging hardware, transducer frequency, and vendor-specific software between centers may also affect the generalizability of models across populations, requiring center-specific recalibration prior to implementation. Finally, regulatory pathways across jurisdictions remain inconsistent for AI-based diagnostic and procedural guidance tools, and novel interventional applications will require prospective outcome data before regulatory agencies and clinical societies adopt such technologies. These practical and regulatory considerations, in addition to addressing the methodological validity concerns outlined above, must be considered before AI-assisted IVUS analysis can be implemented across centers.

## Conclusions

Overall, the evidence gathered in this work indicates that current AI systems are already achieving reasonably good levels of performance in terms of lumen and vessel segmentation on automated coronary IVUS analysis, but on tasks related to stent analysis and tissue characterization, they are somewhat less competent. However, the variations in design, reference standard, and outcome reporting in the eight studies included in the current analysis, together with the fact that all were retrospective, meant that the body of evidence was not sufficient to allow quantitative pooling. By applying the GRADE framework, the overall level of certainty in this body of evidence was judged to be low, primarily due to risk of bias in patient selection and outcome reporting and due to differences between trials in terms of AI-based tasks, reference standards, and outcomes.

In conclusion, although AI-assisted IVUS analysis tools appear to have considerable potential as a time-saving aid in the catheterization laboratory, the current evidence base supports their use as an adjunct to, rather than a replacement for, the expert opinion of a human interventional cardiologist. Prospective, multicenter studies using consistent outcome reporting are needed before firm recommendations can be issued.
